# *Phellodendron *and *Citrus *extracts benefit cardiovascular health in osteoarthritis patients: a double-blind, placebo-controlled pilot study

**DOI:** 10.1186/1475-2891-7-16

**Published:** 2008-05-20

**Authors:** Julius Oben, Ebangha Enonchong, Shil Kothari, Walter Chambliss, Robert Garrison, Deanne Dolnick

**Affiliations:** 1 Laboratory of Nutrition & Nutritional Biochemistry, Department of Biochemistry, University of Yaounde I, Cameroon; 2 Gateway Health Alliances, Inc, 4769 Mangels Blvd, Fairfield, CA, 94534, USA; 3 University of Mississippi, University, MS, 38677, USA; 4 Next Pharmaceuticals, 360 Espinosa Road, Salinas, CA, 93907, USA

## Abstract

**Background:**

The objective of this clinical study was to assess the potential benefit of a dietary supplement, NP 06-1, on cardiovascular protective properties in overweight and normal weight adults diagnosed with osteoarthritis of the knee.

**Methods:**

An 8-week, placebo-controlled, randomized, double-blind study was conducted with four groups, comparing the effects of NP 06-1 to placebo in overweight and normal weight subjects diagnosed with primary osteoarthritis of the knee. NP 06-1 (a combination of two botanical extracts; *Phellodendron amurense *bark and *Citrus sinensis *peel) or matching placebo was given in a dose of two capsules (370 mg each) twice daily. The outcome measures reported are lipid levels, weight, BMI, blood pressure and fasting glucose. Analyses of variance were used to compare changes of physiological measures over the trial period and between groups.

**Results:**

Eighty (80) subjects were enrolled and 45 subjects completed the study. No serious adverse events were reported. NP 06-1 administration was associated with a general improvement in lipid levels. Both the overweight and normal weight treatment groups had significant reductions in triglycerides and LDL-cholesterol, as well as a significant increase in HDL-cholesterol compared to their respective control groups.

Overall there were decreases in blood pressure in both overweight and normal weight treatment groups compared to respective placebo groups. There was also a significant decrease in fasting glucose levels in the overweight treatment group compared to the start of the study and to the overweight placebo group. There was no change in fasting blood sugar for the normal weight groups.

Both overweight and normal weight treatment groups lost a significant amount of weight compared to their respective placebo groups. The overweight treatment group lost an average of 5% body weight after 8 weeks, which was associated with a significant loss in BMI over time.

**Conclusion:**

In this pilot study NP 06-1 had a beneficial effect on cardiovascular risk factors; namely lipid levels, blood pressure and fasting glucose levels. Administration of NP 06-1 was also associated with weight loss.

## Background

Osteoarthritis, the most common form of arthritis, is characterized by degradation of articular cartilage which manifests as joint pain followed by reduced mobility. The origin of the disease is unknown but obesity, joint injury, metabolic diseases, bone and joint malfunctions, genetic factors and age have been implicated. Therapies for osteoarthritis include weight control, physiotherapy and pharmacological agents. Conventional drug treatments include analgesics, anti-inflammatory agents, disease modifying therapies, hyaluronic acid, intra-articular glucocorticoids and topical analgesic/anti-inflammatory agents [1]. More recently there has been a focus on nutritional support. Recent systematic reviews highlight the scientific evidence for potential nutritional and herbal preparations for those with osteoarthritis [2,3].

A systematic review of the literature covering obese subjects diagnosed with osteoarthritis of the knee concluded that osteoarthritis related disability could be significantly improved with a loss of over 5.1% body weight [4]. Besides osteoarthritis related disability, increased body weight is associated with higher incidence of diabetes, hypertension and cardiovascular disease. This array of potential disease risks associated with excess weight has been termed metabolic syndrome. Individuals with three or more of the components of central obesity (excess fat mainly in the abdominal area), hyperinsulinemia, dyslipidemia and hypertension are considered to have metabolic syndrome. Treatment involves lifestyle changes such as diet and exercise, as well as intervention with prescription drugs [5].

The subject of this study, NP06-1 (Flavoxine™/Citrofen™, Next Pharmaceuticals, Inc., Salinas, CA), is a proprietary product consisting of a blend of extracts of *Phellodendron amurense *tree bark and *Citrus sinensis *(orange) peel standardized to berberine and polymethoxylated flavones (PMFs), respectively. NP06-1 was formulated with the goal of combining the beneficial effects of both the phellodendron and orange peel extracts. There are indications in the literature, as well as preliminary unpublished studies conducted by Next Pharmaceuticals, that this combination might reduce inflammation as well as have beneficial effects on lipid levels.

The objective of this clinical study was to study the effects of NP 06-1 compared to placebo on biomarkers related to cardiovascular health. Both overweight and normal weight subjects were studied to determine whether or not there might be a difference in benefits to these two groups. The effects of NP06-1 compared to placebo in the management of joint pain and mobility caused by osteoarthritis of the knee will be the subject of another publication.

## Methods

### Study Design

The clinical trial was designed as a placebo-controlled, randomized, double-blind study with four groups. Forty overweight and forty normal weight subjects were enrolled into either treatment or placebo groups, with twenty subjects in each group as shown in Table 1. Subjects were recruited via advertisements at the University of Yaounde I Teaching Hospital, the Djongolo Baptist Hospital and through the public media. The IRB of the Faculty of Medicine and Biomedical Sciences of the University of Yaounde I in Cameroon approved the study and all subjects provided informed consent. Dr. Julius Oben, Associate Professor of Nutritional Biochemistry at the University of Yaounde I, Cameroon was the principal investigator.

**Table 1:** Study Groups.

**Group**	**BMI Category**	**Treatment**	**Subjects**
**OP**	Overweight*	Placebo	20 (13)
**OT**	Overweight*	Active	20 (14)
**NP**	Normal weight†	Placebo	20 (7)
NT	Normal weight†	Active	20 (11)

*BMI 25 kg/m^2 ^– 40 kg/m^2^

† BMI 18.9 kg/m^2 ^– 24.9 kg/m^2^

The number of subjects initially enrolled is listed; with the number that completed the study in parenthesis.

To be included in the study, participants needed to be men or women, 25 to 60 years of age, diagnosed with primary osteoarthritis of the target knee using the American College of Rheumatology guidelines [6] by the treating physician and confirmed by the clinical investigator, and with a BMI between 25 kg/m^2 ^to 40 kg/m^2 ^(Overweight Groups) or 18.9 kg/m^2 ^to 24.9 kg/m^2 ^(Normal Weight Groups).

The exclusion criteria were: morbid obesity BMI > 40 kg/m^2^, diagnosed with rheumatoid arthritis, joint replacements in any one of the knees, unable to walk without assistance, enrollment in another clinical study in the past 6 months, pregnancy, active infection, autoimmune disease, AIDS, HIV, active hepatitis, active malignancy, or diabetes requiring daily insulin management.

### Administration and Dosage

NP 06-1 is a proprietary product of Next Pharmaceuticals, Salinas, California. It is a blend of *Phellodendron amurense *Rupr. [Rutaceae] tree bark extract standardized to a minimum of 50% berberine and *Citrus sinensis *(L.) Osbeck [Rutaceae] peel extract standardized to a minimum of 30% polymethoxylated flavones (PMF). Berberine and PMFs were chosen as representative chemical constituents because they have demonstrated biological activity. Next Pharmaceuticals has licensed US Patent Nos. 6,184,246 and 6,987,125 obtained by the USDA for actions described by PMFs. NP 06-1 is sold under the trade names Flavoxine™ and Citrofen™.

Subjects were allocated into groups using a random number table and instructed to take two NP 06-1 capsules (370 mg formula per capsule) or matching placebo (identical red two-piece hard shell capsules) with food in the morning and at night (4 capsules per day) for a total of 8 weeks.

Subjects were instructed to avoid taking analgesics (a 5 day wash-out period prior to enrollment) or cholesterol lowering medications (a 30 day wash-out period prior to enrollment) during the study and to stay with their normal exercise and diet regimens.

### Study Variables

The study variables were biomarkers of cardiovascular health: weight, BMI, triglycerides, total cholesterol, HDL-cholesterol, LDL-cholesterol, systolic/diastolic blood pressure and fasting glucose.

### Data Collection

Blood samples (8 ml) were collected by venous puncture at time 0, 4 and 8 weeks. Subjects were requested to come to the study center on the mornings of each site visit after a 12-hour fast. The concentrations of triglycerides, HDL-cholesterol, LDL-cholesterol and fasting glucose, were measured using commercial diagnostic kits (triglyceride Infinity, EZ HDL™ cholesterol, EZ LDL™ cholesterol, Glucose Trinder) from SIGMA Diagnostics. Body weight was determined in 12-hour fasted subjects using a Tanita™ scale and height was measured using a stadiometer.

### Safety Assessment

Subjects were given three emergency telephone numbers to contact during the conduct of the study, if they had any adverse events or other concerns related to the study. Each subject was interviewed during site visits to solicit information on possible adverse effects they might have encountered. Participants were instructed to inform the investigator, if the reason for dropping out of the study was due to adverse effects.

### Statistical Analysis

An analysis of variance model, using repeated measures, was used to compare changes of physiological measures over the trial period and between groups. All statistics were 2-tailed and significance was set at p < 0.05.

## Results

Eighty (80) subjects were enrolled in the study and randomized into four groups designated OP (Overweight Placebo), OT (Overweight Treatment), NP (Normal weight Placebo) and NT (Normal weight Treatment) (Table 1). Forty five (45) subjects completed the study and the reasons given by the dropouts are given in Table 2. The dropouts were fairly evenly distributed among the groups, with the exception of Group NP which lost nearly twice as many participants as the other groups. The majority of Group NP dropouts cited no improvement in their condition as their reason for dropping out. There is no indication that their demographics were different from those who stayed in the study. No serious adverse events were reported by any of the dropouts or by those who completed the study.

**Table 2:** Reasons given by subjects for dropping out of the study

**Group**	**No. Subjects**	**Reason for drop out**
**OP**	2	Reported no improvement in their condition
	1	Nausea and vomiting; Malaria attack
	1	Moved out of town
	3	No reason given
		
**OT**	1	Reported improvement as too slow
	1	Tested positive for hepatitis
	1	Moved out of town
	3	No reason given
		
**NP**	7	Reported no improvement in their condition
	1	Nausea
	1	Started weight management program
	1	Started fasting and stopped treatment
	3	No reason given
		
**NT**	1	Malaria attack
	1	Nausea
	1	Started fasting and stopped treatment
	6	No reason given

Baseline lipid levels for the overweight and normal weight groups were in the normal range. The average levels for total cholesterol were 194.5 to 197.3 mg/dl, HDL-cholesterol 63.6 to 69.8 mg/dl, LDL-cholesterol 89.3 to 123.6 mg/dl and triglycerides 86.0 to 124.8 mg/dl. Baseline blood pressure levels for the overweight and normal weight groups were in the upper limits of the normal range, with average levels for systolic blood pressure from 126.6 to 135.8 mmHg and those for diastolic blood pressure from 81.2 to 84.7 mmHg. Baseline fasting blood sugar values for the groups averaged 56.9 to 113.8 mg/dl, also in the normal range.

### Weight/BMI

In Group OT, the subjects lost a significant amount of weight and their BMI was reduced compared to the start of the study (p < 0.001 at 4 and 8 weeks) (Table 3). They lost an average of 2.5 kg (5.5 pounds, 3.1% of body weight) after 4 weeks and an average of 4.2 kg (9.2 pounds, 5.1% of body weight) after 8 weeks. There were smaller, but significant, changes in weight and BMI from baseline for Group OP. Comparisons of weight loss between overweight groups revealed that the treatment group lost on average 3.7 times as much weight as the placebo group at 8 weeks (9.2 pounds versus 2.5 pounds) (p < 0.001). However, in comparison, the changes in BMI's between the two overweight groups were not significant (Table 4).

**Table 3:** Body Weight, BMI.

**Group**	Time 0	4 weeks	8 weeks	%Δ0–4 wks	%Δ0–8 wks
**OP**					
Weight (kg)	85.0	-1.14 ± 0.96	-1.15 ± 1.30	-1.3*	-1.4*
BMI (kg/m^2^)	31.1	-0.42 ± 0.59	-0.42 ± 0.32	-1.3*	-1.4*
**OT**					
Weight (kg)	81.7	-2.49 ± 1.09	-4.19 ± 1.24	-3.1*	-5.1*
BMI (kg/m^2^)	31.7	-0.94 ± 0.11	-1.59 ± 0.11	-3.0*	-5.1*
**NP**					
Weight (kg)	67.1	-0.27 ± 0.60	-0.76 ± 0.34	-0.4	-1.1*
BMI (kg/m^2^)	24.0	-0.10 ± 0.05	-0.28 ± 0.04	-0.4	-1.2*
**NT**					
Weight (kg)	67.0	-0.51 ± 1.08	-1.18 ± 1.29	-0.8	-1.8*
BMI (kg/m^2^)	24.8	-0.18 ± 0.05	-0.42 ± 0.08	-0.7	-1.7*

*Significant P values < 0.05.

OP = overweight placebo, OT = overweight treatment, NP = normal weight placebo and NT = normal weight treatment.

Values at the start of the study (Time 0) listed in the first column are means of data from individuals. Mean changes after 4 weeks and 8 weeks are listed in the second and third columns as ± Standard Deviation. A negative value indicated a decrease in value and a positive number represents an increase in value. %Δ values are percent mean change in value between time 0 and either 4 or 8 weeks. Values* are significant P-value comparisons (< 0.05) between initial values at Time 0 and either 4 weeks or 8 weeks.

**Table 4:** Inter-Group Analysis Comparing the Paired Treatment to Placebo Groups.

	**Group OP vs. Group 0T**		**Group NT vs. Group NP**	
	**T = 4 weeks**	**T = 8 weeks**	**T = 4 weeks**	**T = 8 weeks**

Weight	p < 0.01	p < 0.001	p < 0.05	p < 0.01
BMI	NS	NS	NS	NS
Cholesterol	p < 0.001	NS	NS	NS
HDL	NS	p < 0.001	p < 0.05	p < 0.05
LDL	NS	p < 0.01	NS	p < 0.01
Triglycerides	NS	p < 0.001	p < 0.01	p < 0.01
Systolic BP	NS	p < 0.05	p < 0.05	NS
Diastolic BP	p < 0.05	p < 0.001	p < 0.05	p < 0.001
Glucose	p < 0.001	p < 0.001	NS	NS

P-value comparisons are between overweight placebo (OP) and overweight treatment (OT) groups and between normal-weight placebo groups (NP) and normal-weight treatment (NT) groups at 4 and 8 weeks. NS = not significant.

In Group NT there was a significant decrease in weight and BMI at 8 weeks (p < 0.05) compared to baseline (Table 3). At 8 weeks, this group lost an average of 1.18 kg (2.6 pounds, 1.8% of body weight). Group NP also had a significant loss of weight and BMI after 8 weeks compared to the start of the study (p < 0.01). Comparisons of weight loss between normal weight groups revealed that the treatment group lost on average 1.5 times as much weight as the placebo group at 8 weeks (2.6 pounds versus 1.7 pounds) (p < 0.01). There was no significant difference in change in BMI's between the normal weight treatment and placebo groups (Table 4).

### Total Cholesterol

For Group OT, there were significant decreases in total serum cholesterol levels at 4 weeks (15.6%) and 8 weeks (21.6%) compared to baseline (both p < 0.001) (Table 5). For Group OP, there were smaller, but significant decreases in total serum cholesterol levels at 4 weeks (7.2%) and 8 weeks (8.0%) compared to baseline (p < 0.01 and p < 0.05, respectively). Subjects in the treatment group had a 2.7 times greater average decrease in total cholesterol in comparison to the placebo group. However the individual data was highly variable and the change in Group OT compared to that in Group OP was not significant (Table 4).

**Table 5:** Lipid Levels.

**Group**	Time 0	4 weeks	8 weeks	%Δ 0–4 wks	%Δ 0–8 wks
**OP**					
Cholesterol	195.7	-13.30 ± 12.20	-14.80 ± 16.83	-7.2*	-8.0*
HDL	69.8	0.86 ± 0.95	-0.87 ± 5.72	1.2	-1.3
LDL	89.3	-12.64 ± 16.49	-13.01 ± 16.89	-13.8*	-14.2*
Triglycerides	124.8	-7.85 ± 10.5	-4.58 ± 7.82	-6.3*	-3.7*
**OT**					
Cholesterol	194.5	-31.6 ± 20.68	-43.45 ± 20.96	-15.8*	-21.7*
HDL	69.7	3.37 ± 10.64	8.33 ± 13.25	4.8	11.8*
LDL	105.9	-32.99 ± 23.61	-47.47 ± 22.24	-31.0*	-44.6*
Triglycerides	118.4	-16.52 ± 21.78	-21.57 ± 22.97	-13.9*	-18.1*
**NP**					
Cholesterol	197.3	-9.83 ± 12.91	14.88 ± 13.37	-4.8	-7.3*
HDL	63.6	-0.36 ± 6.91	4.3 ± 4.88	-0.6	6.8
LDL	123.6	-8.82 ± 17.36	-18.02 ± 14.38	-7.1	-14.5*
Triglycerides	86.0	-3.27 ± 2.38	-5.83 ± 8.30	-3.8*	-6.8
**NT**					
Cholesterol	197.0	-14.77 ± 15.51	-19.45 ± 16.26	-7.3*	-9.7*
HDL	67.3	0.50 ± 4.58	2.63 ± 2.96	0.74	3.9*
LDL	116.0	-12.80 ± 15.86	-19.51 ± 16.89	-11.0*	-16.8*
Triglycerides	88.7	-12.33 ± 6.50	-12.82 ± 6.03	-13.9*	-14.5*

*Significant P values < 0.05.

OP = overweight placebo, OT = overweight treatment, NP = normal weight placebo and NT = normal weight treatment.

Values at the start of the study (Time 0) listed in the first column are means of data from individuals. Mean changes after 4 weeks and 8 weeks are listed in the second and third columns as ± Standard Deviation. A negative value indicated a decrease in value and a positive number represents an increase in value. %Δ values are percent mean change in value between time 0 and either 4 or 8 weeks. Values* are significant P-value comparisons (< 0.05) between initial values at Time 0 and either 4 weeks or 8 weeks.

For Group NT there were significant decreases in total serum cholesterol at 4 weeks (7.3%) and 8 weeks (9.7%) compared to baseline (both p < 0.01). For Group NP there was a significant decrease at 8 weeks (7.3%) but no significant change after 4 weeks (Table 5). There were no significant differences between the normal weight groups.

### LDL-Cholesterol

For Group OT, there were significant decreases in LDL-cholesterol levels at 4 weeks (31.0%) and 8 weeks (44.6%) compared to baseline (both p < 0.001). For Group OP, there were smaller, but significant decreases at 4 weeks (13.8%) and 8 weeks (14.2%) compared to baseline (both p < 0.05) (Table 5). Subjects in the treatment group had an average of 3.1 times greater decrease in LDL in comparison to the placebo group at 8 weeks (p < 0.01).

For Group NT there were significant decreases in LDL-cholesterol levels at 4 weeks (11.0%) and 8 weeks (16.8%) compared to baseline (p < 0.05 and 0.01, respectively). For subjects in Group NP there was a significant decrease in LDL at 8 weeks (14.5%; p < 0.05); with no significant change after 4 weeks (Table 5). Comparison of normal weight groups showed a significant difference between treatment and placebo at 8 weeks (p < 0.01).

### HDL-Cholesterol

For Group OT, there was a significant increase in HDL-cholesterol levels at 8 weeks (11.8%) compared to baseline (p < 0.05). There was no significant change at 4 weeks. For Group OP, there were no significant changes in HDL (Table 5). Subjects in the treatment group had an average of 9 times greater increase in comparison to the placebo group at 8 weeks (p < 0.001).

For Group NT there was a significant increase in HDL-cholesterol levels at 8 weeks (3.9%) compared to baseline (p < 0.05). There were no significant changes in HDL levels compared to the start of the study in Group NP. Comparison of normal weight groups showed a significant difference between treatment and placebo at 4 and 8 weeks (both p < 0.05) (Table 4).

### Triglycerides

For Group OT, there was a significant decrease in plasma triglyceride levels at 4 weeks (13.9%) and 8 weeks (18.1%) compared to baseline (both p < 0.05). Group OP also had a significant decrease in triglyceride levels at 4 weeks and 8 weeks compared to baseline (both p < 0.05) (Table 5). However there was a greater decrease in the treatment group in comparison to the placebo group at 8 weeks (p < 0.001).

For Group NT, there was a significant decrease in triglyceride levels at 4 weeks (13.9%) and 8 weeks (14.5%) compared to baseline (both p < 0.001). For Group NP, there was a significant decrease in triglycerides at 4 weeks but not at 8 weeks. Comparison of normal weight groups showed a significant difference between treatment and placebo at 4 and 8 weeks (both p < 0.01).

### Blood Pressure

For Group OT, there was a significant decrease in systolic blood pressure at 4 weeks (3.3%) and 8 weeks (6.0%) compared to baseline (both p < 0.05). There was also a significant decrease in diastolic blood pressure at 4 weeks (8.3%) and 8 weeks (13.1%) compared to baseline (p < 0.01 and p < 0.001, respectively). There were no significant changes in either systolic or diastolic blood pressure in Group OP (Table 6). Comparison of overweight groups showed a significant difference between treatment and placebo in diastolic blood pressure at both 4 and 8 weeks (p < 0.05 and p < 0.001, respectively) and in systolic blood pressure at 8 weeks (p < 0.05) (Table 4).

**Table 6:** Blood Pressure, Fasting Glucose.

**Group**	Time 0	4 weeks	8 weeks	%Δ0–4 wks	%Δ0–8 wks
**OP**					
Systolic BP	130.8	1.31 ± 12.07	2.15 ± 8.66	1.0	1.6
Diastolic BP	81.8	-3.46 ± 7.47	-2.31 ± 8.62	-4.2	-2.8
Glucose	113.8	0.14 ± 7.77	-9.64 ± 8.99	0.2	-10.7
**OT**					
Systolic BP	135.1	-4.43 ± 8.66	-8.07 ± 11.13	-3.3*	-6.0*
Diastolic BP	82.7	-6.86 ± 6.98	-10.85 ± 9.00	-8.3*	-13.1*
Glucose	73.4	-8.58 ± 11.28	-17.67 ± 11.69	-9.5*	-19.6*
**NP**					
Systolic BP	127.0	-5.66 ± 9.56	-6.66 ± 8.08	-4.5	-5.3*
Diastolic BP	84.7	-7.55 ± 2.07	-10.50 ± 5.40	-8.85*	-12.39*
Glucose	57.6	-0.18 ± 5.6	-0.02 ± 3.98	-0.3	-0.0
**NT**					
Systolic BP	126.6	2.90 ± 12.60	-2.30 ± 9.21	2.3	-1.8
Diastolic BP	81.2	-7.36 ± 9.34	-9.45 ± 9.96	-9.1*	-11.6*
Glucose	56.9	-0.62 ± 5.83	-3.27 ± 7.59	-0.9	-4.8

*Significant P values < 0.05.

OP = overweight placebo, OT = overweight treatment, NP = normal weight placebo and NT = normal weight treatment.

Values at the start of the study (Time 0) listed in the first column are means of data from individuals. Mean changes after 4 weeks and 8 weeks are listed in the second and third columns as ± Standard Deviation. A negative value indicated a decrease in value and a positive number represents an increase in value. %Δ values are percent mean change in value between time 0 and either 4 or 8 weeks. Values* are significant P-value comparisons (< 0.05) between initial values at Time 0 and either 4 weeks or 8 weeks. The unit of measurement for blood pressure is mmHg and for glucose is mg/dl.

For Group NT, there were no significant changes in systolic blood pressure compared to the beginning of the study. However there were significant decreases in diastolic blood pressure at 4 weeks (9.1%) and 8 weeks (11.6%) compared to baseline (p < 0.05 and p < 0.01, respectively). For Group NP, there was a significant decrease in systolic blood pressure at 8 weeks (p < 0.05) but not at 4 weeks. This group had significant decreases in diastolic blood pressure at both 4 and 8 weeks (p < 0.001 and 0.01, respectively). Comparison of normal weight groups showed a significant difference between treatment and placebo in diastolic blood pressure at both 4 and 8 weeks (p < 0.05 and p < 0.001, respectively). Comparison of Groups NT to NP also showed a significant difference in systolic blood pressure at 4 weeks (p < 0.05), but not at 8 weeks (Table 4).

### Fasting Glucose

In Group OT, fasting glucose levels decreased significantly at 4 weeks (9.5%) and 8 weeks (19.6%) compared to baseline (p < 0.05 and p < 0.001, respectively) (Table 6). There were no significant changes in fasting glucose levels in Group OP. Comparison of overweight groups showed a significant difference between treatment and placebo at both 4 and 8 weeks (both p < 0.001) (Table 4).

In Groups NT and NP, fasting glucose levels did not change significantly from the beginning of the study and there were no significant differences between the two normal weight groups.

## Discussion

NP 06-1 was administered to the trial participants with a dose of 4 capsules (1,480 mg) per day with no significant adverse events reported; an indication of safety.

NP06-1 contains a *Phellodendron amurense *tree bark extract standardized to a consistent amount of berberine. NP06-1 also contains a *Citrus sinensis *(orange) peel which contains bioflavonoids, including polymethoxylated flavones (PMFs). Both berberine and PMFs have demonstrated beneficial effects on lipid levels as well as glucose tolerance.

Berberine has been shown in human clinical studies to have beneficial effects on lipid levels: reducing triglycerides and LDL-cholesterol and in one study increasing HDL-cholesterol [6,7]. Berberine may affect lipid levels by increasing expression of hepatic low density lipoprotein receptor [8]. In a hamster model, citrus PMFs caused significant reductions in total cholesterol ranging from 19 to 27%; and in very low density cholesterol (VLDL-C) and low density cholesterol (LDL-C), ranging from 32 to 40% [9].

NP 06-1 generally improved lipid levels in this study, which is consistent with literature reports for berberine and PMFs. In this study, the overweight and normal weight treatment groups had significant reductions in triglycerides and LDL-cholesterol compared to their matched control groups. For HDL-cholesterol, there was a significant increase compared to placebo for both treatment groups. The greatest change in lipid levels was observed for LDL in the overweight treatment group, which decreased by an impressive 44.6%. These results indicate that NP06-1 may have a beneficial effect on cardiovascular risk factors. High triglycerides, total cholesterol and LDL-cholesterol levels, along with low HDL-cholesterol levels, have been established as indicators of risk for cardiovascular disease [10].

Another risk factor for cardiovascular disease is high blood pressure [11]. NP06-1 caused significant decreases in blood pressure in both overweight and normal weight treatment groups compared to the start of the study and compared to their respective placebos. There was also a significant decrease in fasting glucose levels in the overweight treatment group compared to the start of the study and to the overweight placebo group. There was no change in fasting blood glucose for either normal weight group.

Both overweight and normal weight treatment groups lost a significant amount of weight compared to the start of the study and to their respective placebo groups. The overweight treatment group lost an average of 5.1% body weight after 8 weeks. Weight loss has previously been reported to decrease fasting glucose levels [12]. Thus weight loss may have influenced the decrease in fasting glucose levels observed in this study. The weight loss appears to be due to NP06-1 as participants were instructed not to change their exercise or diet regimens. Further, berberine has been shown to reduce body weight and to improve glucose tolerance. A mechanism for this is suggested to be an increase in AMP-activated protein kinase activity in adipocytes [13].

PMFs have also been shown to decrease insulin levels and improve glucose tolerance in hamsters [14]. Research is ongoing on the potential antiatherogenic effects of citrus flavonoids [15,16].

## Conclusion

In a placebo-controlled pilot clinical study, NP06-1 offered several potential health benefits in normal and overweight subjects with osteoarthritis of the knee. These potential benefits include significant improvements in cardiovascular risk factors; namely lipid levels and blood pressure. There appears to have been additional benefits to the overweight group compared to the normal weight group in decreases in fasting glucose. Treatment-induced weight loss was also observed.

## Competing interests

This study was sponsored by Next Pharmaceuticals. Mr Garrison was President and CEO. Dr. Chambliss and Mr. Kothari were compensated as consultants. Miss Dolnick is employed by Next Pharmaceuticals.

## Authors' contributions

All authors were involved in the design of the study, as well as analysis and interpretation of the data. The study was carried out by Dr. JO, the Principal Investigator and Miss EE, his assistant.
